# A dietary pattern rich in fruits and dairy products is inversely associated to gestational diabetes: a case-control study in Iran

**DOI:** 10.1186/s12902-021-00707-8

**Published:** 2021-03-04

**Authors:** Abazar Roustazadeh, Hamed Mir, Sima Jafarirad, Farideh Mogharab, Seyed Ahmad Hosseini, Amir Abdoli, Saiedeh Erfanian

**Affiliations:** 1 Department of Biochemistry, School of Medicine, Jahrom University of Medical Sciences, Jahrom, Iran; 2 Department of Advanced Medical Sciences and Technologies, School of Medicine, Jahrom University of Medical Sciences, Jahrom, Iran; 3 Nutrition and Metabolic Diseases Research Center, Clinical Research Institute, Ahvaz Jundishapur University of Medical Sciences, Ahvaz, Iran; 4 Research Center for Non-communicable Diseases, Jahrom University of Medical Sciences, Jahrom, Iran; 5 Department of Nutrition, School of Allied Medical Sciences, Ahvaz Jundishapur University of Medical Sciences, Ahvaz, Iran; 6 Department of Obstetrics and Gynecology, Jahrom University of Medical Sciences, Jahrom, Iran

**Keywords:** Pregnancy, Gestational diabetes, Dietary pattern, Body mass index

## Abstract

**Background:**

Gestational diabetes mellitus (GDM) causes many problems for mother and her neonate. A healthy diet plays an important role in preventing GDM. This study aimed to investigate the relationship between major dietary patterns and the GDM.

**Methods:**

386 healthy and 306 GDM pregnant women (total 693) completed this case-control study. Basic information and anthropometric indices were recorded, and a food frequency questionnaire was completed. For extracting major dietary patterns, the principal component analysis was performed. Multivariable logistic regression models were used to examine whether specific dietary patterns are associated to the GDM.

**Results:**

Four dietary patterns were identified: “fruits and dairy products”, “red meat and plant-based foods”, “snacks and high-fat foods” and “carbohydrate-rich foods”. Among these major extracted dietary patterns, “fruits and dairy products” showed an inverse association to the GDM (odds ratio adjusted for confounders: 0.50, confidence interval: 0.284–0.882, p-trend = 0.019, for highest vs. lowest quartile).

**Conclusions:**

It seems using a healthy dietary pattern such as “fruits and dairy products” may decrease GDM risk.

**Graphical abstract:**

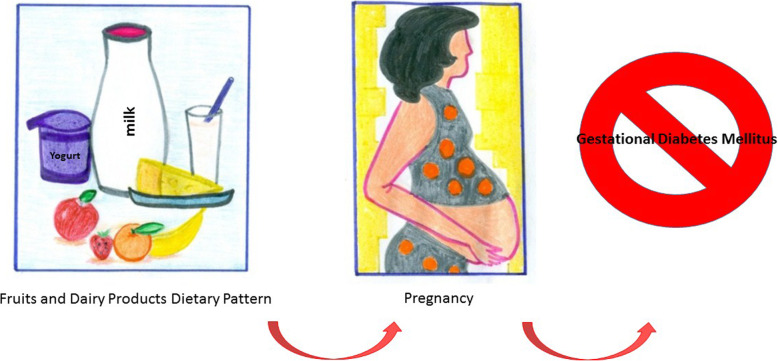

## Background

Gestational diabetes mellitus (GDM) is a disorder during pregnancy, defined as glucose intolerance in women who have never had diabetes so far [1]. The GDM causes many complications during pregnancy or after childbirth for mother and her baby [2]. In 2017, it was reported 1 in 7 births was affected by the GDM worldwide [3]. GDM prevalence in Asia is estimated by 11.5 % [4]. In Iran, a report showed a range from 1.3 to 18.6 % in GDM prevalence [5]. However, GDM incidence in the population who live in urban area is similar to developed countries [6].

Obesity and a family history of diabetes are major risk factors for the GDM [7]. Obesity affects insulin resistance, so it may elevate GDM risk [8]. Having a diet with more energy intake from foods including sweet snacks and fats lead to overweight, obesity [9], or type 2 diabetes mellitus (T2DM) [10].

Previous studies focused on the relationship among nutrients or single foods and GDM [11, 12]. A dietary pattern shows the type of a diet and various foods which people habitually consume [13] which is related to the culture and social factors. It is a better index to assess the relationship between a disease and a diet in epidemiologic studies [14]. Some studies showed rich dietary patterns in the whole grains, fruits, vegetables, and nuts such as prudent and Mediterranean dietary patterns have an inverse relationship with the GDM [15, 16]. Besides, animal-based dietary patterns may increase GDM risk [17]. It seems a diet rich in fruit, vegetables, whole grains, and fish and low in red and processed meat, refined grains, and high-fat dairy can decrease in GDM risk [18].

There are some differences in the type of dietary patterns between Western and Asian countries due to different cultures. A study in China showed the relationship between the traditional Chinese diet and GDM risk [19]. It was an interesting finding, because it showed a traditional diet may lead to an increase in GDM risk. Although conflicting results showed there is no relationship between dietary patterns and GDM risk in the Asian countries [20].

Iran is an Asian country with various cultures and ethnicities. These different cultures may lead to various dietary patterns. There are limited studies about the relationship between dietary patterns and the GDM in Asia and Iran. There are conflicting results due to different cultures [19–21]. Therefore, we tried to find a relationship between major dietary patterns and the GDM in Jahrom, a city in the South of Iran.

## Methods

### Participants

This study is a case-control study conducted in Jahrom, a city in Fars province, South of Iran. Pregnant women who were referred to Motahari and Honari hospitals participated in the study. A simple sampling method was used for selecting subjects. The inclusion criteria for the case and control groups were pregnant women from 24th to 28th weeks of gestation, age ranges of 18–40 years, no history of diabetes or the GDM, having no chronic diseases (such as cardiovascular diseases, chronic renal disease, liver, and gastrointestinal diseases, hypo or hyperthyroidism, and severe anemia), singleton pregnancy and no weight loss program before pregnancy. Abortion, preeclampsia, eclampsia, and incomplete questionnaires were considered exclusion criteria in case and control groups. One-step procedure was used to diagnose the GDM in the case group. In this procedure, the oral glucose tolerance test was done. Blood glucose was measured after 8–12 h of fasting. Then, pregnant women were given 75 g of oral glucose, and blood glucose was measured again after one and two hours. The GDM was diagnosed if any blood glucose values were equal or more than 92 mg/dL, 180 mg/dL, and 153 mg/dL in fasting, 1 and 2 h after oral glucose consumption, respectively [22].

This study was a 1:1, case-control study. The required sample size was calculated assuming the relationship between major dietary patterns and the GDM, due to a study in Asia, which found a traditional dietary pattern was related to the odds of GDM (the highest quartile of traditional dietary pattern compared to the lowest quartile; odds ratio = 2.92) [19]. The sample size was calculated with Fleiss et al.. method [23], using an online OpenEpi calculator (version 3) for case and control studies [24]. By considering 4 % of controls exposed, the sample size was determined by 305 subjects in each case and control group (90 % power of study and a 95 % level of confidence). At first, 565 GDM and 634 healthy pregnant women were invited to study. 435 GDM and 493 healthy pregnant women accepted the invitation. Due to the inclusion criteria, 383 in the case and 454 in control have remained. 67 subjects in the case and 49 subjects in control refused participation in the study, and 721 pregnant women participated (316 in the case and 405 in control). Twenty-eight subjects (10 in the case and 18 in control) were excluded due to incomplete questionnaires. Finally, 693 subjects (306 in the case and 387 in control) were included in the analysis.

### General data collection

Subjects completed a general questionnaire about their age, weight before pregnancy, number of children, active smoking before pregnancy (yes, no), level of education, and socioeconomic status (very low, lower medium, medium, high) [25]. The socioeconomic status was determined by asking about income. Then, the participants were divided into very low, lower medium, medium, and high. Level of education was categorized into illiterate or primary school, high school or diploma, college, and university. The level of physical activity was determined by the short form of international physical activity questionnaire [26]. The metabolic equivalent was determined which used to divide participants into sedentary, very low, low, medium, and hard activity levels. In the hospital, a trained technician with an accurate scale measured the weight of pregnant women monthly and recorded the weight and date of health card visit. Therefore, the health card was used to record the trend of weight gain during pregnancy. Pre-pregnancy body mass index (BMI) was calculated by reported pre-pregnancy weight in kilograms divided by the height in squared meters.

### Dietary assessment

A food frequency questionnaire (FFQ) was used to estimate dietary intakes with 168 food items. The reliability and validity of FFQ were shown in a study in the Iranian adult population which used twelve 24-hours dietary recalls to compare nutrients intake from FFQ [27]. This questionnaire was completed via a face-to-face interview with the trained nutritionists. Participants reported their intake frequency of an intended food in FFQ during last year, basis on daily, weekly, monthly, or annual frequency of intake. To increase dietary assessment accuracy, interviewers used a booklet which showed them the image of portion size. After completing FFQ, the frequency of each food item was converted into the daily intake. We used household measurements to convert each food to the gram. Besides, food intakes were adjusted for total energy intake by residual method [28]. Data were entered into SPSS (version 17.0, SPSS Inc., Chicago, IL, USA) software to extract dietary patterns.

### Dietary pattern extraction

**Fig. 1:**
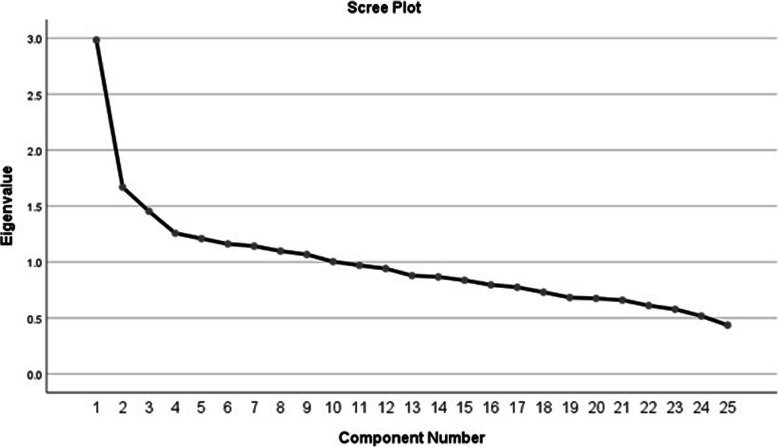
The scree plot of eigenvalues; the point at which the graph becomes horizontal, shows the number of factors to be kept

Considering foods similarity, they were categorized into related food groups. Twenty-five food groups were included analysis (Table 1). Principal component analysis (PCA) was used to find major dietary patterns due to energy-adjusted foods intake. This analysis method is widely used which is an adaptive descriptive data analysis tool. Besides, it provides information regarding the maximum number of factors [29]. Kaiser-Meyer-Olkin (KMO) and Bartlett’s tests were used to assess factor analysis suitability. KMO ranges between 0 and 1, and a minimum value for good factor analysis is 0.6. Besides, Bartlett’s test should be significant (*p* < 0.05) [30]. The sampling sufficiency of components was approved by the KMO test > 0.67. Moreover, inter-correlation of components was confirmed using Bartlett’s test of sphericity < 0.001. We used a factor eigenvalue to decide several factors to retain. Besides, we used the scree plot which involves plotting each value. Dietary patterns were determined due to eigenvalue > 1.2 and scree plot examination (Fig. 1). Orthogonal rotation (varimax) was applied to simplify data interpretation. Factor loading more than 0.3 was chosen to find more relationships between food groups and dietary patterns. The dietary pattern was named using principal food groups in each pattern. A score was assigned to all participants considering adherence to each dietary pattern. Quartiles of factor scores were determined due to total participants and considered in the further analysis [31].

**Table 1:** Rotated factor loading in four major dietary patterns^a^

	Food groups (Items)	Fruits and dairy products	Red meat and plant- based foods	Snacks and high-fat foods	Carbohydrate- rich foods
1	**Fresh fruits** (cantaloupe, melon, watermelon, pear, apricot, cherry, apple, peach, nectarine, plum, fig, grape, kiwi, grapefruit, orange)	0.685			0.330
2	**Fruit Juices** (grapefruit juice, orange juice, apple juice, cantaloupe juice, canned fruit)	0.608			
3	**High fat dairy products (**high fat milk, cocoa milk, strained yogurt, high fat yogurt, traditional ice cream, other ice creams**)**	0.602			
4	**Low fat dairy products** (low fat milk, skim milk, low fat yogurt, doogh (traditional yogurt drink))	0.554			
5	**Creamy dairy products** (creamy cheese, creamy yogurt, cream, butter)	0.396			
6	**Olive products** (fresh olive, olive oil)	0.370	0.325		
7	**Raw vegetables** (lettuce, tomato, cucumber, leek, parsley, scallion, basil, carrot, raw spinach, bell pepper)		0.594		
8	**Whole cereals** (barley, oatmeal, corn (all types))		0.499		
9	**Nuts and seeds** (peanuts, walnuts, almonds, pistachios, hazelnuts, sunflower seeds)		0.484		
10	**Onion, garlic and cabbage** (raw onion, garlic, pepper (red and black), cabbage (all types)		0.465		
11	**Dried fruits (**dried figs, raisins, dates, dried berries, dried apricots, dried peaches)		0.451		
12	**Red meats (**beef, Lamb meat, minced lamb, sheep’s tongue, broth)		0.406		
13	**Legumes** (lentil, pinto bean, pigeon pea, broad bean, mung bean, soybean)		0.465	0.332	
14	**Pickles and sauces** (ketchup, traditional pickles, sour lemon juice)		0.373	0.417	
15	**Salt and salty snacks** (salt, salty popcorn, chips)			0.590	
16	**Cakes and sweets** (biscuit, cracker, tea cake, creamy cake, other cakes, sugar, tablet sugar, honey)			0.587	
17	**Organ Meats** (tripe, sheep brain, leg of lamb, heart and liver of lamb, sausage)			0.487	
18	**Fats and oils** (animal fat, mayonnaise, hydrogenated oil, cooking oils, margarine)			0.466	
19	**White meats** (chicken, fish (all types), shrimp)			0.447	
20	**Traditional breads** (Lavash, Barbari, Sangak, Taftoon)				0.557
21	**Cooked vegetables** (squash, stewed vegetables, eggplant, zucchini, celery, boiled carrot, fried onion, cooked spinach, mushroom, turnip)				0.475
22	**Other cereals and starch sources** (rice, pasta, potato, French fries, vermicelli, traditional vermicelli (Reshte))				0.420
23	**White breads** (baguette bread, toast, wheat flour)				-0.313
24	**Cheese and whey** (cheese (all types), traditional whey (Kashk))				
25	**Tea and coffee** (tea (all types), coffee (all types))				
	Variance explained (%)	11.93	6.67	5.81	5.03

^a^Factor loading < ± 0.3 was not shown

### Statistical analysis

Kolmogorov-Smirnov tested the normality of data. Quantitative variables were compared between the case and control groups by independent sample *t*-test and categorical variables by the Chi-squared test. One-way analysis of variance was used to compare the mean of quantitative variables among quartile groups of the factor scores of dietary patterns. Tukey test was applied as a post-hoc analysis. Non-parametric tests were used in case of non-normal distribution. The logistic regression model was used to find the relation of independent variables and extracted dietary patterns with gestational diabetes. In this analysis, the lowest quartile was considered as a reference. Besides, this test was used to eliminate the effect of confounders. The odds ratio (OR) was determined with a 95 % confidence interval (CI). Stratified analysis was applied to assess a relationship between the dietary pattern and the GDM stratified by pre-pregnancy BMI levels using Mantel-Haenszel test. All analyses were done using SPSS version 17.0 (version 17.0, SPSS Inc., Chicago, IL, USA). A *p*-value less than 0.05 was considered a significant level.

## Results

This study was started from November 2018 which completed in September 2019. Total 721 pregnant women (405 healthy and 316 GDM) participated in the study. Twenty-eight subjects (18 in control and 10 in case) were excluded because of incomplete FFQ. At last, 693 subjects (387 in control and 306 in case) were included in the analysis.

Table 2 shows the comparison between healthy and the GDM pregnant women for demographic, anthropometric, and socioeconomic variables. Subjects age with the GDM was significantly more than healthy (*p* < 0.001). Besides, pre-pregnancy BMI and weight gain during pregnancy in the GDM subjects were more than healthy ones (*p* < 0.001). There was no difference between two groups for smoking and job. Chi-square analysis showed a difference in socioeconomic status, physical activity, education, and the number of pregnancies between two case and control groups (*p* < 0.001).

**Table 2:** The comparison of baseline characteristics between healthy pregnant women and suffered from gestational diabetes mellitus (GDM)^a^

variables	GDM (*N* = 306)	Healthy (*N* = 387)	*p*-value**
**Age**	32.7 ± 5.3	28.30 ± 5.4	**< 0.001**
**Weight, pre-pregnancy**^b^	69.7 ± 12.4	63.4 ± 11.6	**< 0.001**
**BMI, pre-pregnancy**^b^	27.3 ± 4.5	24.8 ± 4.2	**< 0.001**
**Weight gain**^b^	9.1 ± 5.2	7.4 ± 4.8	**< 0.001**
**Education**			**< 0.001**
Illiterate or primary school	44.2 %	55.8 %	
High school and diploma	59.5 %	40.5 %	
College and university	37.1 %	62.9 %	
**Number of pregnancies**			**< 0.001**
First	30.1 %	69.9 %	
Second	46.4 %	53.6 %	
Third and more	53.5 %	46.5 %	
**Gender of neonate**			
Male	46.5 %	53.5 %	0.249
Female	43.6 %	56.4 %	
**Job**			
Housewife	43.7 %	56.3 %	0.984
Employee	43.7 %	56.3 %	
Self-employed	46.2 %	53.8 %	
**Socioeconomic status**			
Very low	55.4 %	44.6 %	**< 0.001**
Lower medium	27.4 %	72.6 %	
Medium	53.1 %	46.9 %	
High	20.0 %	80.0 %	
**Active smoker**			
Yes	51.6 %	48.4 %	0.242
No	43.6 %	56.4 %	
**Activity**			
None	40.0 %	60.0 %	
Very low	72.4 %	27.6 %	**< 0.001**
Low	29.0 %	71.0 %	
Medium	54.5 %	45.5 %	
Hard	38.1 %	61.9 %	

*BMI* Body mass index

^**^Chi-squared test for categorical and Independent *t-*test for quantitative variables

^a^Data are shown as mean ± standard deviation for quantitative variables and percent for categorical variables

^b^Non-normal distribution, Mann-Whitney test

Four major dietary patterns were extracted using PCA. Due to the scree plot, four dietary patterns with eigenvalues higher than 1.2 were selected as major dietary patterns (Fig. 1). These dietary patterns were: “fruits and dairy products”, “red meat and plant-based foods”, “snacks and high-fat foods,” and “carbohydrate-rich foods”. Table 1 shows the factor loading of food groups in each extracted dietary pattern. The cumulative variance of four dietary patterns was 29.45 %. The “fruits and dairy products” pattern consisted of 11.93 % of the variance and contained fresh fruits, fruit juices, olive, and dairy products. The “red meat and plant-based foods” pattern consisted of 6.67 % of the variance and mainly contained vegetables, cereals, nuts, legume and red meats. The “snacks and high-fat foods” pattern consisted of 5.81 % of the variance, and including cake and sweets, salty snacks (such as chips), organ meats, fats and oils, and white meats. The “carbohydrate-rich foods” pattern consisted of 5.03 % of variance and mainly including traditional bread, other cereals, and starch sources (such as potato), and cooked vegetables.

Age, anthropometric indices, number of pregnancies, job, level of education, and socioeconomic status were compared among the quartiles of factor scores of dietary patterns (Table 3). Smoking and physical activity level were not shown because we did not find any significant value. Age of subjects was different among the quartiles of “fruits and dairy products”, also “snacks and high-fat foods” dietary patterns. In the fourth quartile of “snacks and high-fat foods,“ pregnant women had a lower age than the first quartile. However, BMI of pre-pregnancy and weight gain during pregnancy were not different among each dietary pattern quartiles. There was a significant difference in education and the number of pregnancies among the quartiles of “snacks and high-fat foods” dietary pattern. Besides, there was a significant difference and a trend near significance for socioeconomic status and level of education among the quartiles of “fruits and dairy products” dietary pattern (Table 3).

**Table 3:** Comparison of age, anthropometric indices, number of pregnancies, and socioeconomic factors among quartiles (Q1-Q4) of major extracted dietary patterns

	Fruits and dairy products		Red meat and plant- based foods		Snacks and high-fat foods		Carbohydrate-rich foods	
	Q1	Q4	Q1	Q4	Q1	Q4	Q1	Q4
**Age**	30.5 ± 5.7	29.2 ± 5.3	29.5 ± 5.9	30.2 ± 5.6	31.4 ± 5.4	28.3 ± 5.8	29.8 ± 5.4	30.0 ± 5.6
P^*^	**0.010**		0.255		**< 0.001**		0.615	
**Weight pre- pregnancy**	66.4 ± 13.1	66.1 ± 11.6	64.8 ± 11.3	66.9 ± 10.6	67.2 ± 11.8	63.2 ± 13.1	66.9 ± 12.1	66.6.1 ± 13.6
P^**^	0.915		0.344		0.135		0.815	
**BMI pre- pregnancy**	25.6 ± 4.8	25.8 ± 4.4	25.9 ± 4.4	25.8 ± 4.6	25.8 ± 4.4	26.4 ± 4.5	25.8 ± 4.2	26.4 ± 4.9
P^**^	0.996		0.844		0.178		0.235	
**Weight gain**	8.6 ± 5.1	8.1 ± 4.9	8.2 ± 5.1	8.0 ± 4.6	8.2 ± 4.6	8.2 ± 5.3	8.2 ± 5.2	8.1 ± 5.1
P^**^	0.120		0.768		0.997		0.530	
**Education**								
Non or primary	30.6 %	19.8 %	25.4 %	26 %	18 %	35.3 %	26.7 %	31.4 %
High school and diploma	39.3 %	43 %	46.8 %	38.7 %	34.9 %	42.2 %	45.3 %	37.8 %
College and university	30.1 %	37.2 %	27.7 %	35.3 %	47.1 %	22.5 %	27.9 %	30.8 %
p^***^	**0.061**		0.086		**< 0.001**		0.202	
**Number of pregnancies**								
First	33.1 %	33.5 %	29.7 %	28.3 %	24.3 %	39.5 %	32.9 %	27.3 %
Second	32 %	37.6 %	32 %	34.7 %	37.6 %	34.3 %	30.6 %	39.5 %
Third and more	34.9 %	28.9 %	38.4 %	37 %	38.2 %	26.2 %	36.4 %	33.1 %
p^***^	0.459		0.331		**0.004**		0.184	
**Job**								
House wife	89 %	87.2 %	89.6 %	88.4 %	82.6 %	91.3 %	92.4 %	88.3 %
Employee	8.1 %	11.6 %	6.9 %	9.8 %	15.1 %	7.6 %	5.2 %	11.1 %
Self-employment	2.9 %	1.2 %	3.5 %	1.7 %	2.3 %	1.2 %	2.3 %	0.6 %
p^***^	0.533		0.357		0.134		**0.003**	
**Socioeconomic status**								
Very low	20.2 %	6 %	20.8 %	10.8 %	9.4 %	17.3 %	17.5 %	13.5 %
Lower medium	27.2 %	45.2 %	27.2 %	37.7 %	38.8 %	32.7 %	33.7 %	30.6 %
Medium	52.6 %	47 %	50.9 %	49.1 %	48.2 %	48.8 %	46.4 %	54.7 %
High	0 %	1.8 %	1.2 %	2.4 %	3.5 %	1.2 %	2.4 %	1.2 %
p^***^	**< 0.001**		0.181		**0.059**		0.456	

Data are presented as mean ± standard deviation for quantitative variables and percent for categorical variables

^*^One-way analysis of variances

^**^Non-normal distribution, Kruskal–Wallis test

^***^Chi-squared test

Results showed “fruits and dairy products” pattern could inversely predict GDM (*p*-trend = 0.003). After adjustment for age, BMI of pre-pregnancy, weight gain during pregnancy, energy intake, socioeconomic status, education, physical activity, and the number of pregnancies, this finding was significant again (*p*-trend = 0.019). These factors were adjusted, because they were different between the cases and controls. The second quartile of “snacks and high-fat foods” dietary pattern predicted GDM (*p* = 0.017), and *p*- trend was near significant (*p* = 0.068). However, after adjustment for confounders, this significant relation was not seen (Table 4).

**Table 4:** The odds ratio (95% confidence interval) of gestational diabetes mellitus based on quartile of dietary pattern score

Dietary pattern			Model 1			Model 2			Model 3		
		N	OR	CI	*P*-trend^*^	OR	CI	*P*-trend^*^	OR	CI	*P*-trend^*^
Fruits and dairy products	Q1	175	1	-	**0.003**	1	-	**0.004**	1	-	**0.019**
	Q2	176	0.623	0.383-1.013		0.668	0.399-1.120		0.575	0.327-1.011	
	Q3	176	0.507	0.308-0.833		0.511	0.303-0.862		0.472	0.267-0.835	
	Q4	172	0.478	0.29-0.785		0.488	0.289-0.825		0.500	0.284-0.882	
Red meat and plant-based foods	Q1	177	1	-	0.634	1	-	0.798	1	-	0.950
	Q2	169	0.964	0.591-1.571		0.869	0.519-1.455		0.901	0.512-1.585	
	Q3	173	0.827	0.508-1.346		0.754	0.451-1.263		0.659	0.373-1.163	
	Q4	174	0.742	0.456-1.209		0.712	0.422-1.200		0.660	0.372-1.173	
Snacks and high-fat foods	Q1	175	1	-	**0.068**	1	-	0.750	1	-	0.672
	Q2	184	1.794	1.108-2.905		1.889	1.139-3.135		1.709	0.988-2.957	
	Q3	162	1.203	0.728-1.988		1.325	0.777-2.262		1.153	0.642-2.068	
	Q4	172	0.800	0.484-1.321		1.082	0.617-1.897		0.931	0.498-1.741	
Carbohydrate-rich foods	Q1	168	1	-	0.569	1	-	0.592	1	-	0.559
	Q2	186	0.960	0.588-1.567		0.997	0.592-1.679		1.065	0.606-1.872	
	Q3	167	1.408	0.852-2.329		1.542	0.905-2.629		1.743	0.970-3.130	
	Q4	172	1.387	0.843-2.283		1.982	1.129-3.481		1.795	0.984-3.277	

Model 1: crude model; Model 2: adjusted for age, the body mass index of pre-pregnancy, weight gain during pregnancy, and total energy intake; Model 3: additionally adjusted for socioeconomic status, level of education, physical activity and number of pregnancies

*OR* odds ratio, *CI* confidence interval

^*^The logistic regression model, the lowest quartile as reference

Because pre-pregnancy BMI may reflect some problems during pregnancy such as the GDM, we adjusted this effect by stratified analysis. Pre-pregnancy BMI was divided into four levels: underweight (< 18.5), normal (18.5–25), overweight (25–30), and obese (> 30). Due to the median of score, the score of factors of “fruits and dairy products” dietary pattern was divided into two levels. By adjusting pre-pregnancy BMI effect, the odds of GDM were lower in the higher median of “fruits and dairy products” dietary pattern (Mentel-Haenszel common OR: 0.580, CI: 0.413–0.810). It seems pre-pregnancy BMI is a confounding factor, but not interacting.

## Discussion

The relationship between the GDM and anthropometric indices and social factors was investigated. Besides, PCA was used to identify major dietary patterns and their association with the GDM.

“Fruits and dairy products” showed an inverse relationship with the GDM. Less prediction of GDM was seen in the third and fourth quartile of this pattern. Also, this inverse relationship was significant after adjustment for the effect of confounders. This pattern’s main foods were all kinds of dairy products, fresh fruits and fruit juices and all kinds of olive products. The GDM is characterized as glucose intolerance in pregnancy. It develops when a woman cannot produce enough insulin during pregnancy. Fruits and olives contain phytochemicals such as polyphenols. Polyphenols may affect glucose homeostasis by several mechanisms. These mechanisms include stimulating insulin secretion, controlling the digestion of carbohydrate and absorption of glucose, controlling the release of glucose from the liver and finally activating insulin receptors [32]. Therefore, dietary patterns, which are rich in fruits and olives, such as the Mediterranean diet, may help pregnant women to prevent the GDM. Although “fruits and dairy products” dietary pattern has some differences from Mediterranean diet, it contains Mediterranean diet’s main foods, especially fruits and olives. Some documents showed that dairy products’ consumption is an effective factor in the prevention of T2DM [33]. A study investigated the effect of “dietary approaches to stop hypertension” (DASH) on insulin resistance in the GDM which showed the improvement of fasting blood glucose and serum insulin [34]. A DASH diet is due to whole grains, fruits, vegetables, and low-fat dairy products. Whereas, in “fruits and dairy products” pattern, there are two principal items of DASH diet. All kinds of dairy products were included in this dietary pattern. However, Anue *et al*. confirmed the protective effect of low-fat dairy products and cheese on T2DM incidence [33]. It seems the positive effect of dairy consumption is due to their potential calcium sources [35]. A study showed high calcium intake from diet is related to less incidence of the GDM [36]. Besides, dairy products contain whey protein as an important nutrient. Whey protein may improve hyperglycemia by several mechanisms including stimulation of insulin secretion and the action of incretin hormones such as glucagon-like polypeptide-1 and gastric inhibitory peptide [37].

“Snacks and high-fat foods” dietary pattern consists of more unhealthy foods. We did not find any relationship between this unhealthy dietary pattern and the GDM. However, the crude model was near to being significantly associated, but no significant value was observed after adjustment for confounders. The main foods in this dietary pattern included fats and oils, organ meats, white meats, legumes, sauces, cakes and sweets, salt and salty snakes which all of them except legumes were unhealthy. Although “snacks and high-fat foods” were similar to Western dietary pattern, legumes might neutralize unhealthy foods’ effect in this dietary pattern. In consistent to our study, two studies in Asia showed no relationship between Western dietary pattern and the GDM [20, 38]. However, two other studies found that using Western dietary pattern increased GDM risk [19, 21]. Besides, we did not find any relationship between “red meat and plant-based foods” and “carbohydrate-rich foods”, with GDM. There are some cultural factors, may affect eating habits which lead to different metabolic events [39]. Besides, diet composition heterogeneity may be another reason for no relationship among these dietary patterns with GDM [40].

### Strength and limitation

The present study’s strength is an almost large sample size to extract the dietary patterns and homogeneity of subjects for ethnicity. This study has some limitations. Firstly, FFQ is a semi-quantitative questionnaire and depends on the food list which may cause some errors in food intake estimation, although we used a 168-food item food frequency questionnaire to extract dietary patterns which is a comprehensive questionnaire. Another limitation is using multiple questionnaires which made the mothers bored. Recall bias is also a limitation of study, because mothers were asked to report the food intake during the previous year. Besides, there is not a cause and effect association in a case-control study.

Iran contains different cultures, and it would be suggested for future research to study GDM risk concerning culture and dietary patterns in well-designed prospective studies.

## Conclusions

Dietary pattern is an effective factor in the incidence of many chronic diseases. Finding an appropriate dietary pattern such as “fruits and dairy products” could help pregnant women to prevent the GDM. Further prospective studies and clinical trials are needed to confirm the results of this study.

## Data Availability

The datasets generated and analyzed during the current study are available from the corresponding author on reasonable request.

## Abbreviations

BMI: Body mass index
CI: Confidence interval
DASH: Dietary approaches to stop hypertension
FFQ: Food frequency questionnaire
GDM: Gestational diabetes mellitus
KMO test: Kaiser-Meyer-Olkin test
PCA: Principal component analysis
T2DM: Type 2 diabetes mellitus
